# Governing socio-ecological transition in an industrial city: public institutions’ prominence and the risk of a top–down process

**DOI:** 10.3389/fsoc.2026.1899843

**Published:** 2026-09-30

**Authors:** Lidia Greco

**Affiliations:** Department of Political Sciences, University of Bari Aldo Moro, Bari, Italy

**Keywords:** governance, industrial cities, public institutions, socio-ecological transition, Taranto

## Abstract

This article examines the governance of the socio-ecological transition in the industrial city of Taranto (Italy) as it strives to move beyond its historical dependence on the steel industry and towards a more sustainable model of development. Based on the qualitative evidence, the article shows the protagonism of local public institutions, namely, the Puglia Region and the Municipality of Taranto. Through a mix of ideational, political, and economic mechanisms, local public institutions have adopted a post-industrial economic model and articulated a vision for Taranto’s transition aligned with a green growth perspective. While positively valuing the unprecedented effort toward the diversification of the area’s economy and society, the local public-led governance raises concerns regarding administrative capacity, the extent of social participation, and the extent to which alternative perspectives are incorporated to achieve its ambition of a just socio-economic transformation toward climate neutrality. The article seeks to deepen understanding of urban and regional transformation driven by the ecological transition in industrial areas and offers an original contribution to the literature on Taranto by moving beyond the predominantly industrial perspective to examine the broader territorial and urban governance arrangements, as well as the socio-economic dynamics underpinning the transition.

## Introduction

Considerations over the biophysical limits of economic growth and the challenge of reconciling economic development, social equity, and environmental protection, first articulated in two seminal works, *The Limits to Growth* (Meadows et al., 1972) and the Brundtland Commission’s *Our Common Future* (World Commission on Environment and Development, 1987), remain central to contemporary debates on ecological transition. The traditional assumption that economic, and especially industrial, growth has historically caused harmful emissions and, more generally, the depletion of natural resources has increasingly been complemented by the recognition that environmental degradation itself, such as loss of biodiversity and extreme weather events, limits economic growth in a two-way relationship. The coronavirus disease 2019 (COVID-19 pandemic and earlier), the 2008 financial crisis have highlighted the fragility of existing economic systems and the limitation of growth models based on short-term expansion; these crises have reinforced the need for more resilient and sustainable pathways capable of addressing the challenges posed by economic and social vulnerabilities but also by climate change (Wernli et al., 2023).

Favoring a sustainable socio-economic transition implies, among other things, being able to pursue its principles and govern the processes that concretize them. Questions revolve around who acts on sustainable transition and in what ways, by acknowledging that environmental governance, namely, the set of regulatory processes, mechanisms, and organizations through which political actors influence environmental actions and outcomes, includes international organizations, States, corporations, global civil society, and other actors (Bernstein et al., 2009).^1^ It is widely recognized that political commitment to achieve sustainability objectives has emerged significantly at the international level through multilateral agreements, operationalized through action plans such as the United Nations (UN) Agenda 21 connected to the Rio Declaration in 1992, the more recent Paris Agreement in 2015, and the European Union (EU) Green Deal in 2019. Until now, the State features, explicitly or implicitly, centrally in discussions of collective responses to the ecological crisis. The environmental (Duit et al., 2016) or green (Hildingsson et al., 2019) State, for instance, is analyzed for its ecological impacts and the potential role its policies can play in fostering the decarbonization of industry (Hausknost and Hammond, 2020). As stressed by Newell (2026), the need to simultaneously innovate, regulate, consult, redistribute and globalize transitions confronts the State with a series of tensions and trade-offs. The expansion of a large literature on the “just” transition emphasizes the challenge of inequalities as the ecological transition is a profoundly selective process: people, sectors and territories will be differently affected by the desired transformation and, at the same time, have a different capacity to undertake sustainability paths, benefiting from the advantages that “green growth or green economy” can open up (Caprotti and Bailey, 2014; Woehrle, 2010). Beyond technical accounts, ecological transition is fundamentally a socio-cultural and institutional transformation (Askenazy and Didry, 2023; Crivello, 2023).

The examination of the multiscale nature of climate governance has been particularly rich, with a growing body of work examining how cities around the world are facing the sustainability challenge, also through networking, joining global transmunicipal organizations, sharing best practices, and inspiring GHG reduction efforts (Kuramochi et al., 2020; Wei et al., 2021). Specifically, Hickmann (2021) has explored the vertical, horizontal, and transnational entanglement and interconnectedness of cities and local governments to capture better the potential and limitations of urban policymaking contributing to global sustainability. Cities and their governments are well positioned to engage with such issues; however, they also face budgetary and institutional capacity constraints and require support from actors at other levels of government and across societal scales to carry out effective sustainability transitions. Related perspectives, emerging from economic geography, sociological analysis, and labor environmentalism, focus on industrial cities and regions to explain why they face greater difficulties in transitioning toward more sustainable development. Their endeavor is not solely to adapt their economies, often monocultural and reliant on heavy industries (Geng et al., 2019; McKendry, 2018), and their societies to green imperatives, but to reshape them profoundly (Beatty and Fothergill, 2020; Warren et al., 2021). In such cases, defining and governing ecological transition emerges as a critical testing ground.

This article contributes to such debates by investigating the governance of the ecological transition in the industrial city of Taranto (Italy) as it strives to move beyond its historical dependence on the steel industry and toward a more sustainable model of socio-economic development. It attempts to contribute to the understanding of urban and regional transformation, driven by the ecological transition, in industrial areas by examining the governance of possible change. While acknowledging the prominent role of the local public actors through various governance mechanisms, the article also argues that the top-down nature of this process raises concerns regarding administrative capacity, the extent of social participation, and the extent to which alternative perspectives are incorporated to achieve its ambition of a just socio-economic transformation. The article also offers an original contribution to the literature on Taranto by moving beyond the predominantly industrial perspective and labor environmentalism that have characterized previous research, and instead examining the broader territorial and urban governance arrangements and socio-economic dynamics underpinning the transition.

Methodologically, the article has combined quantitative and qualitative techniques. The documentary analysis (involving academic literature, official policy documents, technical reports, and press articles) was used to examine specific, technical issues and context-related information, and was supplemented by qualitative research based on 33 semi-structured interviews with Taranto’s citizens. Further eight interviews with key local observers have been conducted to examine the research topics and their preliminary results. Although not statistically representative, such interviews sought to account for the following main structural variables: gender, age, and school qualification. The topics addressed in the interviews concerned: (a) the meaning of transition and its trajectories in Taranto; (b) the actors involved, their logic of action, their concrete practices; (c) the possible socio-economic consequences of such trajectories; (d) the meaning and the practices to ensure a just transition; (e) personal feelings about the transition. The majority of the interviews were carried out between June 2023 and January 2024; the fieldwork ended in Autumn 2024. Interviews were recorded, transcribed, coded and analyzed using computer-assisted qualitative data analysis software. They highlighted different capacities to understand and possibly navigate the complexity of the changes underway, depending especially on interviewees’ knowledge and reflections on the theme, as well as on direct experiences of change. Despite these differences, all participants shared an awareness of this complexity as well as a widespread aspiration for a decent life and wellbeing. The interviews corroborated the knowledge and understanding the author had developed on the topic. Still, they also brought to light insights that had previously been underestimated, such as the interviewees’ interpretation of transition primarily as a social transition and the importance attributed to social dimensions, including rights in transition, decent work, collective challenge, and so on.

These interviews were conducted as part of a broader research project aimed at providing a deeper understanding of Taranto’s industrial and territorial transition and diversification, and at systematizing the author’s previous long-term research activity in the area. The project also resulted in the publication of a book, entitled Greco (2026), which presents an original sociological investigation into the current socio-economic transformation of Taranto. The choice to analyze the experience of Taranto stems from the fact that it represents an emblematic case that exposes the unresolved challenges surrounding industrial production, public intervention, and the safeguarding of the environment and society. This brings to light fundamental dilemmas that extend well beyond the local context and are shared by many industrial regions worldwide.

The article is structured as follows. After the Introduction, Section 2 accounts for the cities’ growing role in environmental policy and governance and identifies industrial cities as critical testing grounds for ecological transition. Section 3 provides the contextual background to the Taranto case study by tracing the area’s socio-economic evolution and prolonged industrial stalemate that has constrained its more recent development. This section also examines the policy framework and the interventions designed to foster socio-economic diversification, starting from the enactment of a regional law dedicated to the strategic planning of the area. Building on the empirical evidence, Section 4 presents the governance mechanisms underlying the public-led transition in Taranto, while Section 5 reflects on the potential and shortcomings of such a process.

### Governing socio-ecological transition: industrial cities as critical testing grounds

The climate crisis, demanding serious forms of action at all levels of decision-making, needs to be governed, financed, regulated and coordinated, and patterns of production and innovation need to be steered and shaped by policymaking, in a way that is also just and without resentment (Galgoczi, 2022; McCauley and Heffron, 2018; Rodríguez-Pose, 2018). Scholarly work has approached the roles of different players in terms of visions, scope, and strategies in the green transition and, more systematically, the analysis of global governance (Bernstein et al., 2009). Corporations, transnational environmental organizations, civil society organizations, and trade unions have increasingly intersected with the State’s actions in shaping human-environment interactions. However, it remains the only institution capable of making legally binding social rules. As one of the State’s multiple political levels, cities are being increasingly investigated for their crucial role in environmental policymaking and governance, and in addressing this paradigmatic shift (McKendry, 2018). The pursuit of a sustainable, ecological transition, together with the definition of an energy agenda, undoubtedly represents the most recent traits of an urban political turn (Gandy, 2022; Bahers et al., 2022). Contrary to the longstanding view that they represent the antithesis of nature, cities have become key actors in the transition for a variety of reasons. They are, it is argued, where the impacts of climate change are felt most directly and, therefore, where people are most likely to take action to reduce emissions (Evans and Karvonen, 2010; Swyngedouw, 2013). Cities are also active places where mobilizations and alternative views on current forms of globalization, as well as on the objectives of sustainability and social transformation, often emerge (Oscilowicz et al., 2025; Sareen and Lund Waagsaether, 2023).

More specifically, cities and their governments are increasingly recognized as important actors in global sustainability governance, especially for their capacity to formulate pioneering strategies. The 2030 Agenda for Sustainable Development, adopted in 2015, highlights the significance of the local level in attaining sustainable development worldwide (United Nations, 2015). Several networks have emerged to join forces and cooperate for their common interests: for instance, the C40 Cities Climate Leadership Group, the Global Resilient Cities Network, the International Council for Local Environmental Initiatives (ICLEI)—Local Governments for Sustainability—the Association of Southeast Asian Nations (ASEAN) Smart City Network. Thus, while cities are certainly to be considered as sites for creative responses to global problems, for new policy cultures, and as important hubs for innovation, their capacity to address sustainability issues varies considerably, constrained not only in terms of resources but also by their dependence on support from actors operating at other governmental levels. As cities function within multilevel governance dynamics, their ability to adopt and implement sustainable policies is influenced by regional, national, and international institutions. Hickmann (2021) has analyzed the multi-level governance system in which cities are inserted and pointed to three relevant dimensions: the vertical interlinkages of cities with regional and national governments and their involvement in international relations; the horizontal interplay of municipalities with public and private actors in their jurisdictions; and the transnational interplay of cities in networks and alliances.

The challenge of transition is especially relevant for cities and surrounding territories that host industrial, polluting activities in several aspects. First, the transition to a more sustainable economic structure may jeopardize industrial cities and regions, particularly where monocultural economies depend on polluting, hard-to-abate industries. In these territories, where the industrial and broader economic structures largely coincide, an irreversible industrial crisis can trigger broader territorial decline, with serious impacts on employment, economic wellbeing, and demographic trends, among others. Deindustrialization studies have highlighted the localized impacts of factory closures and the crises experienced by deindustrialized communities. These extend beyond the economic sphere and include, among others, the erosion of social cohesion, the weakening of place identities and attachment, demoralization and loss of purpose, and enduring nostalgia (Cowie and Heathcott, 2003; Devine-Wright, 2022); Mah (2012) advanced the concept of wasted places to highlight how certain places become marginal by processes of capitalist development. Confined in the middle-income trap, such areas risk becoming entrenched in the plight of “left-behind places” (McKinnon et al., 2022) and contribute to increased “green discontent” (Rodríguez-Pose and Bartalucci, 2024). Until now, some industrial cities and areas might find themselves in a paradoxical state of affairs in which they undergo the loss of industrial jobs without losing the industry itself and the associated noxious burdens: they therefore live in a context of noxious deindustrialization, a negative-sum game in which industrial job losses and environmental degradation progress simultaneously (Feltrin et al., 2022; Lerner, 2012). Second, industrial cities and areas are likely to be disproportionately affected by the adoption of sustainable transition measures. The environmental justice literature highlights the selective nature of industrial restructuring and of burdens with reference to age, class, sectors and geography. As hinted above, in these places, the profound reshaping of economies and societies to meet green imperatives comes with unpredictable outcomes, underscoring the unique challenges such cities face in the pursuit of sustainability (Lewartowska et al., 2024; Mah, 2025; Smith and Stirling, 2010). Third, a well-established literature on “old” industrial areas in sociology and economic geography has used the concept of path-dependence to explain the difficulties that some industrial areas encounter in adapting to changing economic circumstances (among others, Martin and Sunley, 2006; Hassink, 2005; Hudson, 2005). Supported and fed by specific ‘lock-in’ mechanisms -of functional, cultural and political nature- (Grabher, 1993) and their reciprocal relations, the institutionalization of long-term developmental pathways prevents the system from deviating from the initial conditions (Ebbinghaus, 2005). However, while existing institutions, structures and actors, internal and external to a place, influence its developmental trajectories, change can also occur. The goal of social analysis is also, therefore, to identify agency and mechanisms, such as the concurrence of opportunities and strategies, capable of breaking or sustaining set territorial trajectories. The following section examines the experience of the steel city of Taranto to trace its trajectory of transition toward a more sustainable development model, analyzing both the nature of this transition and the governance that has emerged along the way.

### In search of a metamorphosis: a public-led socio-ecological transition in the city of Taranto

#### A steel city in transition

Located in Southern Italy, the city of Taranto has slightly more than 187,000 residents; considering also its province, more than half a million people live in the area. Long-term economic data show a process of deindustrialization and a parallel process of tertiarization, especially in traditional industries such as commerce and tourism. The number of manufacturing firms declined substantially between 1971 and 2022, falling from 4,400 to just over 2,500 (−42%). As a result, the manufacturing sector’s share of all firms decreased from 26% in 1971 to 7.3% in 2022 (Figure 1).

**Figure 1:**
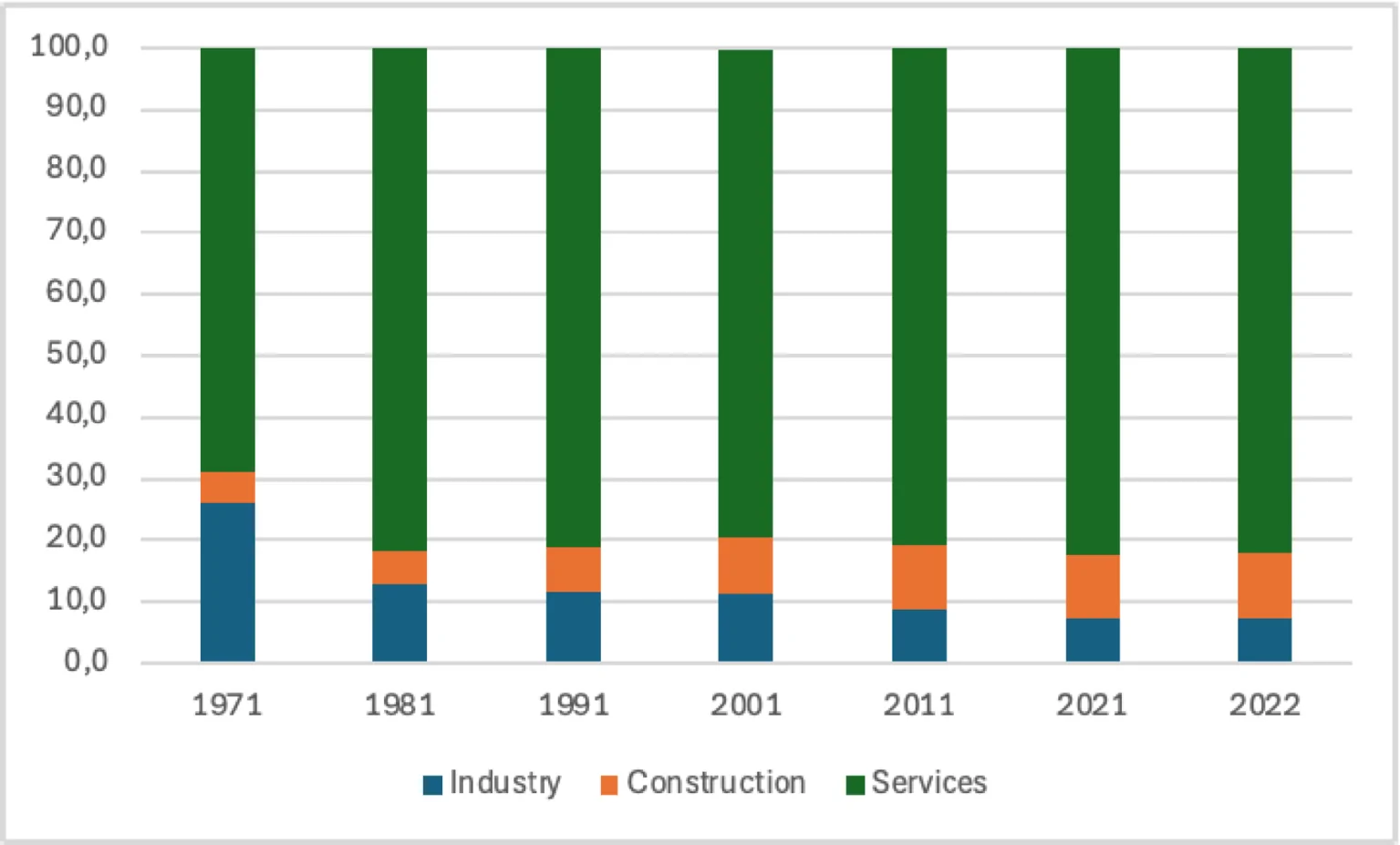
Firms local units by broad economic sectors in the province of Taranto (%). Source: ISTAT, Census of Economic Activity, (various years).

Employment in manufacturing also contracted over the same period (Figure 2). In 2022, the sector employed just over 28.400 workers, accounting for 24.2% of total employment. In 1971, employment in the industry represented approximately 40% of total employment. Nonetheless, the industrialization index^2^ in Taranto is still higher than the regional average (5,1 compared to 4,3).

**Figure 2:**
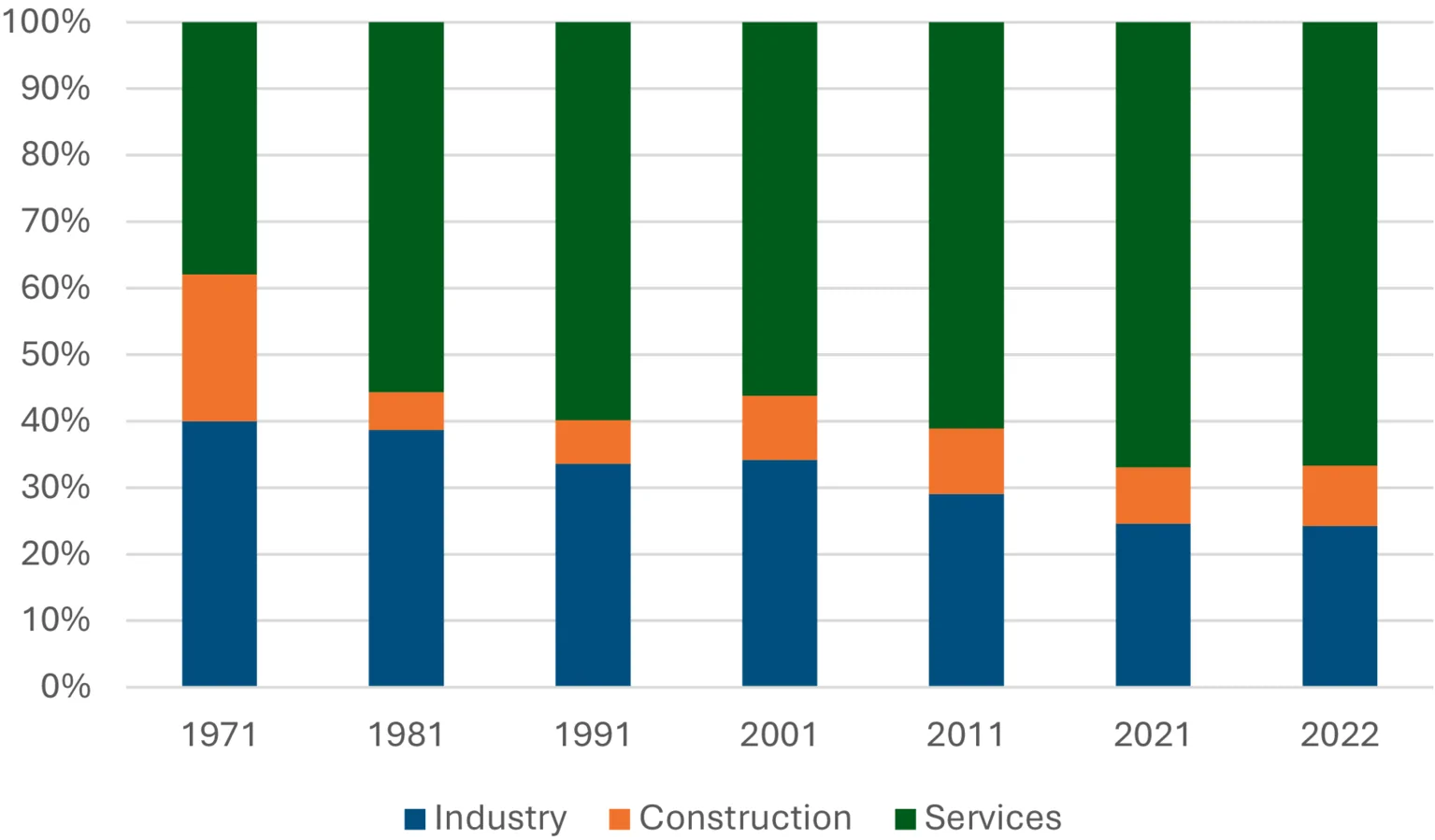
Local unit employment by broad economic sectors in the province of Taranto (%). Source: ISTAT, Census of Economic Activity, (various years).

Taranto is a steel city—home to the largest European integrated steel complex in Europe. Its developmental trajectory has been linked to the settlement of the publicly owned steel plant—Italsider, later ILVA—in the late 1950s as part of a national strategy of industrialization and modernization of Southern Italy. This second industrialization, after the one developed in connection with the presence of the Military base, rapidly became the core of the local economy and identity, employing tens of thousands of direct and indirect workers and generating a path-dependent development pattern. Over time, local entrepreneurship and the political class remained largely tied to the publicly owned industry, limiting diversification and reinforcing an exogenous development model (Greco and Di Fabbio, 2014; Romeo, 2019). The industry underwent several restructuring processes: first in the 1980s, in the context of European-level steel rationalization plans, and then in the 1990s, when the international privatization swept the sector. ILVA was privatized in 1995, becoming part of the Riva Group. The change in ownership produced a substantial organizational discontinuity; the new owner also modified the long-term relationship between the steel plant and the community, unwilling to cultivate stable and committed relationships with Taranto’s workers and citizens (Chiarello and Greco, 2014). In the following years, until 2012, the company underwent periods of growth alternating with more challenging ones. Still, its activity was accompanied by increasing social tensions within the community, which called for stricter environmental regulations for the plant—awareness of the environmental issue in Taranto dates to the very beginning of steel production. As in the rest of Italy, workers were among the main actors behind demands for improved working and environmental conditions during the 1970s. In the following decades, however, extensive industrial restructuring substantially weakened their influence, leading to a marked decline in their bargaining power and public role in the community (Greco, 2021). Prolonged exposure to industrial emissions has indeed produced severe environmental and health impacts on Taranto’s population and on its territory. In 2012, the judicial seizure of parts of the plant for an “environmental and health disaster” made visible a structural, but also naturalized, conflict between the right to health and the right to work (Leonardi, 2023). The State’s subsequent entry into the company property ended the Riva family’s ownership. Since then, the State’s commissioners have pursued three interconnected strategies: ensure the continuation of production, safeguard employment levels, and seek a new private investor for the business, which in 2017 was identified as the steel multinational ArcelorMittal. Its presence in Taranto, however, lasted less than 5 years, from 2018 to 2024. Blaming the steel multinational for its unwillingness to invest effectively in the local plant, the State took back control of the company and once again sought a potential investor to carry out the plant’s decarbonization, which had become imperative following the adoption of the EU Green Deal. It is important to stress that public ownership of the steel plant has not prevented the ongoing environmental damage, as reflected in the repeated extensions of the Integrated Environmental Authorization.

The existence of a polycrisis, consisting of environmental degradation, a public health emergency combined with declining industrial activity and social distress, made it more urgent than ever to question the very existence of the steel plant; it further increased the urgency of the area’s transformation and diversification process, which had been slowly taking shape over time, as documented in a previous study (Greco and Di Fabbio, 2014). In this process of change, local public institutions, namely, the Puglia Region and the Municipality, have become much more active than before to address the area’s transition.^3^ This has occurred within a multiscale architecture that includes national and European institutions and is involved in a comprehensive strategic policy framework to steer the achievement of climate neutrality by 2050.

#### Local strategic planning in a multiscale framework: navigating socio-ecological transition

“Change is now”: this is what is written in the Strategic Plan “Taranto Near Future” (Asset-Regione Puglia, 2019, p. 72). Contemplated by the Puglia Region law 2018, n. 2,^4^ this is the first Plan committed to promoting and supporting the area of Taranto’s path change toward sustainability. Not only is such a plan important for its political value, but it is also a framework orienting the future choices, interventions, and public expenditure in a multiscale framework (Figure 3).

**Figure 3:**
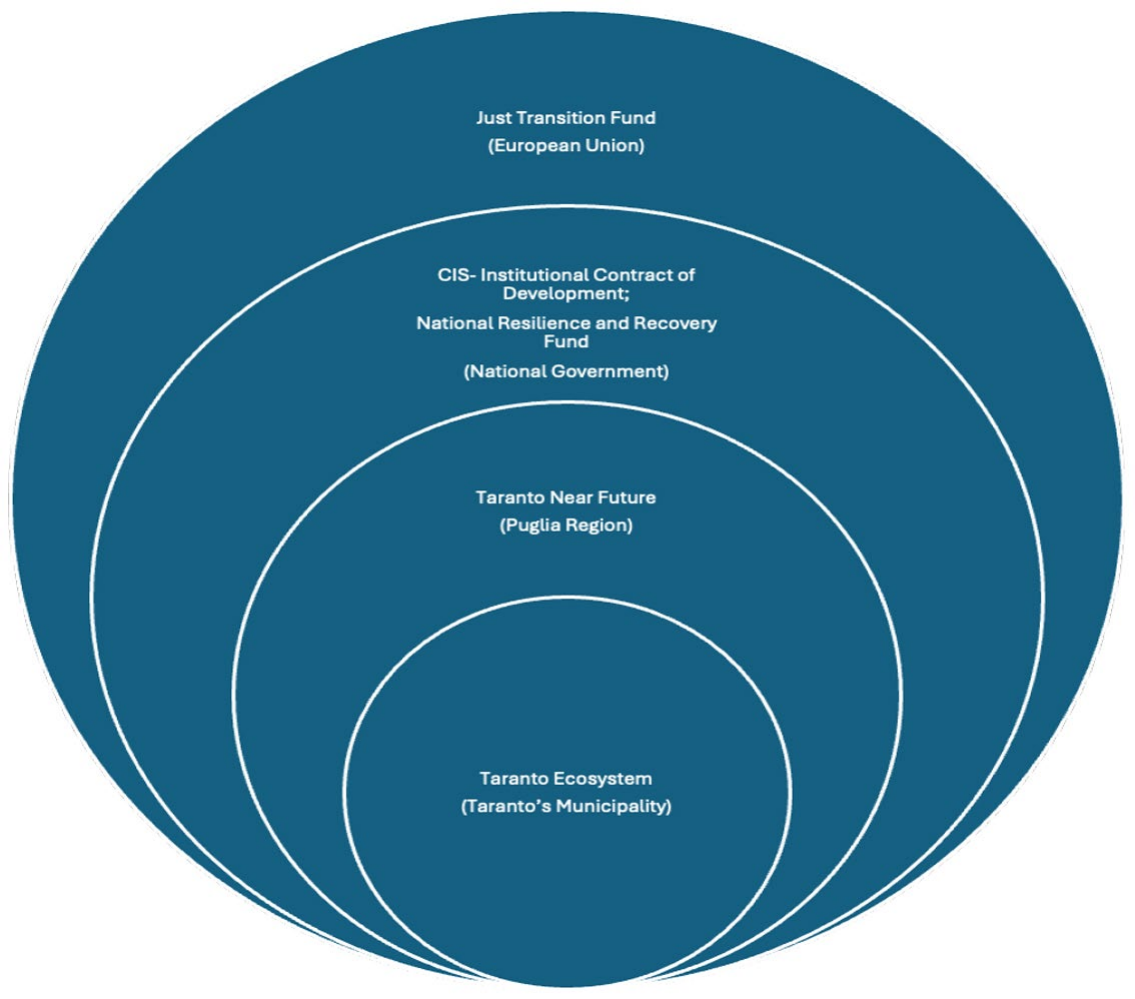
Public programs of intervention in Taranto. Source: Author’s own elaboration.

The Strategic Plan identifies three pillars on which Taranto should base its future economic development. One pillar concerns the Environment: it focuses on remediating polluted areas, promoting renewable energy, and eliminating coal from the steel cycle. The so-called blue economy represents the second pillar: it implies the relaunch of traditional economic activities connected to the sea, from mussel cultivation to boat construction and repair, but also the expansion of Port activities,^5^ to strengthen Taranto’s role in the Mediterranean area. The third area of economic development is that of culture and creativity, through the valorization of Taranto’s archaeological and cultural heritage, the development of creative industries, support for sports activities, and, more generally, the promotion of tourism and innovation in symbiosis with research and training. To enhance these pillars, local institutions have identified six operational axes that aim to overcome the two major issues deemed to hinder the development of Taranto: a poorly diversified economic fabric and a low quality of life. In addition to this Strategic Plan, in 2019, the Municipal Administration also launched an ecological, economic, and energy transition plan called “*Taranto Ecosystem,*” based on the objectives of the United Nations 2030 Agenda. In short, in a scenario envisaging the downsizing of the steel sector and its decarbonization, the aspiration is for a shift from manufacturing to a service-oriented, environmentally sustainable economy, from a steel monoculture to polyphonic development. A further dimension of change pertains to urban development, with the start of the administrative procedure for the city’s General Urban Plan.

Policies and practices of local institutions are nested in national and supranational scales. Started in 2012 in an emergency phase and immediately after the judiciary’s intervention on the steel plant, the “Contratto Istituzionale di Sviluppo (CIS)”, in 2015, has become the single National programming tool for all interventions specifically addressed to Taranto (see infra). National resources have also reached Taranto through the National Recovery and Resilience (NPRR) Plan.^6^ Finally, together with Sulcis Iglesiente (in the Region of Sardinia), Taranto and its province are the recipients of the EU Just Transition Fund (JTF) (Greco et al., 2023). As part of the Mechanism for the Just Transition, the JTF is one of the financial instruments envisaged by the European Green Deal for territories expected to be most negatively impacted by the transition toward climate-neutrality.

The documentary evidence provided highlights in Taranto the role of public institutions in a context of multilevel convergence of policies and resources. In the next section, drawing on fieldwork findings, the article discusses the governance mechanisms that enable local public actors to pursue transition strategies and projects, highlighting the opportunities and shortcomings of this institutionally led path to sustainable socioeconomic development.

### Governing the transition through institutional leadership: the mechanisms at play

The vertical alignment of political strategies and narratives across European, national and local institutions, each acting within their respective spheres of competence, is one of the mechanisms that have created the conditions for institutional leadership of sustainable transition in Taranto; this alignment is centered on the shared understanding that the ecological transition offers a promising pathway for the growth of the city. The governance of socio-ecological projects and strategies relied especially on a fruitful, converging political alliance between Regione Puglia and the Municipality of Taranto, whose leading elite belonged to the same political party. Through a special law for Taranto, consultations and partnerships, and dedicated financial resources, the political support of the Puglia Region played a decisive role in shaping local sustainability policy options.

*When considering the actors involved, in addition to the Renzi government, there was also a significant hand from Governor Emiliano; essentially, I consider Melucci Governor Emiliano’s godson.*^7^ *(Int. 1 – Warehouse worker in a private company).*

*Returning to the issue… this is a city that has been governed for years, indeed for some decades now, by the same political camp. This continuity produces significant results in some fields but, overall, one would expect to deliver even more. (Int. 23 – Small entrepreneur).*

*In any case the Region deserves credits for this [Emiliano had the intuition that Taranto needed transformation or would have died]. Over the past few years, starting with the Melucci’s first term as mayor, the Municipality has sensed that the city was entering a phase of transformation and has worked to promote its cultural assets and so on. (Int. 35 – former local politician).*

The adoption of the strategic planning document is another mechanism through which local public actors have reinforced their ideational and political leadership. With it, Regione Puglia has signaled willingness to assume a central role in addressing and implementing the Taranto transition from a medium-to-long-term perspective. Strategic planning is, in fact, an essential tool for the design and implementation of public policies that aim to provide a proactive and stable policy framework in complex situations. It represents a major challenge for public institutions themselves, which cannot limit their role to designing the plan but must also promote, facilitate and implement it. As clearly expressed by a senior public official:

*Because today economic-cultural responses are hardly of individual entity that is, they require a network, a network of initiatives, of synergies, therefore between the different entities… these are models… (Int. 36 – Senior public official).*

A complementary dimension of change concerns the attempt to modify the city’s identity. The use of images such as “Taranto the Spartan city,” “Taranto the capital of the Magna Graecia,” and “Taranto the capital of the Sea” is easy to understand. By selecting certain aspects of its past and attributing to them an original memory that can become a territorial brand and a new form of placemaking, that is, a new narrative, the aim is to reconfigure the collective representation of the city both within the community and externally; if Taranto is to transform its development trajectory, it must first learn to conceive and represent itself differently (Greco, 2026).

Thirdly, local public governments and administrations have been primarily responsible for managing and directing the substantial public resources allocated to Taranto in recent years. This has ensured concrete implementation opportunities. CIS funds, more than 1 billion euros, privileged infrastructural interventions for the port and transport (41% of resources), for the environment and tourism (17%), for the health sector (28%) and for urban and territorial regeneration initiatives (9%). Up to April 2026, the NPRR had allocated 4.3 billion euros to the province of Taranto, of which more than 3 billion was directed to the urban area, supporting infrastructural interventions as well as initiatives in the areas of environmental sustainability, business and employment, health and social inclusion, culture and tourism. With regard to the JTF, of the total allocation of € 20 billion, Italy received over € 1,200 million, 66% of which (approximately € 795 million) was allocated to the Taranto area. As envisaged by the EU, financial resources have been allocated on three priorities (Figure 4): (a) the reskilling and upskilling of workers and the definition of pathways to increase labor market mobility and welfare (26%); (b) the diversification of the local economic fabric (45%); (c) the environmental sustainability of the lands through remediation of polluted areas and industrial sites and energy transition (29%). More specifically, eight operational actions have been defined to pursue them (Table 1), among which actions supporting the development of renewable energies, hydrogen production, research and innovation, employment creation and entrepreneurship, and welfare services. At the time of writing, the implementation phase of these actions has just begun, following initial administrative delays in developing the executive plan. Numerous calls for proposals have been issued, inviting industrial and service firms (or their associations) as well as third sector organizations to apply. No official figures have yet been issued on the uptake of these actions, nor is there any concrete evidence of their impacts. At the local level, however, widespread and converging concerns have been raised about the need to ensure that financial resources provided are used effectively and appropriately. The challenge is precisely to translate policy programs into useful and impactful actions in a situation in which, for instance, welfare and employment measures are called upon to address serious health and environmental problems and declining employment in the industrial sector.

**Figure 4:**
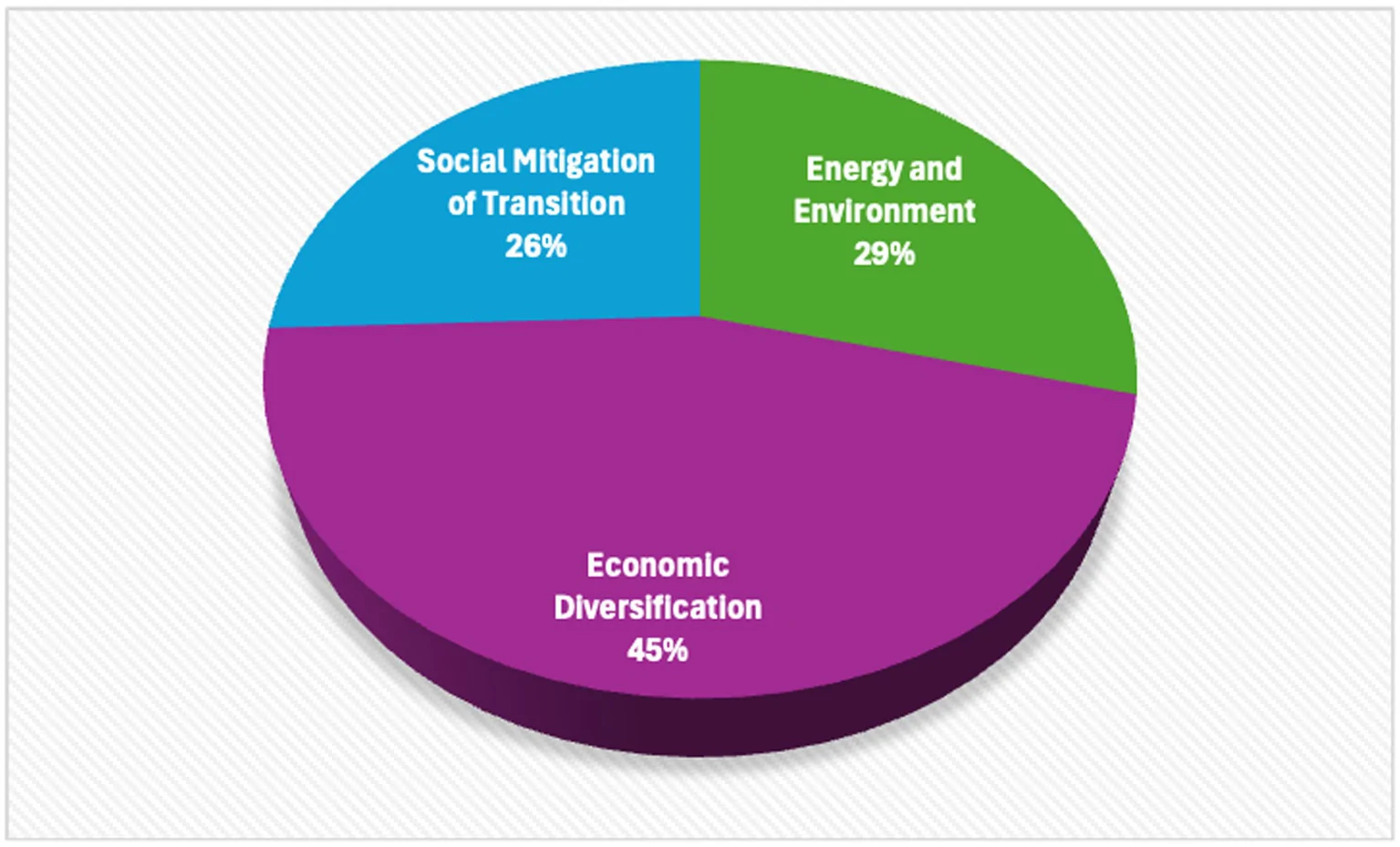
Resources of the JTF (2021-2027)– Program for the Province of Taranto by priority of intervention (%). Source: Department of Cohesion Policy and of the South.

**Table 1:** Resources of the JTF (2021-2027)– Executive Program for the Province of Taranto by action (%).

Actions	Resources (%)
1 Support for renewable energy	6,0
2 Support for the green hydrogen value chain	4,0
3 Support for ecological transition and the protection of natural resources	18,9
4 Support for research projects promoting the transition and diversification of the economy	14,2
5 Strengthening technical support for innovation and economic diversification	13,0
6 Entrepreneurship development and business creation	18,3
7 Support for workers reskilling; strengthening employment services	17,8
8 Provision of care and social services	7,8
Total	100,0

Source: Department of Cohesion Policy and of the South.

### Opportunities and shortcomings of a top–down transition

Taranto’s transition toward a new development model centered on sustainability, initiated by local public institutions, represents an unprecedented shift. If genuinely pursued, the delineated strategy could pave the way for diversifying the local economy, bringing about the structural transformation long sought at the local level and marking a clear break from more than a century of dependence on single industrial sectors—the military apparatus first, the steel industry afterward. Nonetheless, the local institution-led governance of the socio-economic transition also reveals significant shortcomings that warrant further reflection on both its implementation and its capacity to foster a shared vision of the future.

Firstly, the multilevel political convergence of interests that has underpinned local ascendancy began to unravel after the far-right Government led by Giorgia Meloni came to power. Diverging political visions and governing practices increasingly set the center-left coalitions leading the Regional and Municipal administrations at odds with the national Government.^8^ Clear examples of setbacks are the activities of CIS, whose commissioner is appointed by the central Government, and the EU JTF, whose concrete implementation at the local level suffered from the abolition in December 2023 of the Cohesion Agency nationally and the transfer of its functions to the Cohesion Department under the Presidency of the Council of Ministers.^9^

Additionally, interscalarity and multilevel governance require greater coordination and administrative capacity. Although Taranto local actors have shown active engagement and interaction across different institutional levels, the absence of recognized and strong leadership, together with limited formal autonomy, soon became apparent. An overall impression emerged of fragmented interventions across different levels of government, each acting within its own remit, resulting in an implementation process lacking coherence and unity. In such situations, the risk is that different administrative levels reduce long-term planning to a list of projects according to a distributional logic (Bobbio, 2000). A senior public official explains such a danger as follows:

*Because today economic and cultural responses are hardly of an individual institution, that is such responses require a network, a network of initiatives, of synergies, therefore they are the outcome of the work of various institutions… these are the prevailing models…… Resources [financial]are ultimately not the result, they are a means, they serve for a change finalized to new development models. If we look at spending as such, this can determine a growth of income, it can certainly happen in a short-medium period, but once the resources are exhausted nothing will have changed. (Int. 36 – Senior public official).*

Furthermore, in the shift from strategic planning to concrete operational choices, its innovative features have been sacrificed in favor of consolidated administrative routines. For instance, public calls for proposals under some measures of the JTF, such as those addressed to small and medium-sized enterprises (SMEs), have seen the utilization of traditional rather than tailored and place-based tools, such as Pia and mini-Pia.^10^ As indicated, part of the problem lies in the quality of public administrations, their capacity to innovate their modus operandi, and their willingness to value different, also collaborative, practices, especially in Southern Italy (Polverari, 2020). The same civil servant highlights that:

*civil servants must slowly get used to it [working in synergy, involve other institutions] as it is in the case of northern Italy, where collaborative and synergic forms are a little more structured, due to historical factors specific to those territories. So this is crucial because there are so many expectations, but I would not want them to turn into illusions because you know disillusion generates lack of hope and mistrust.* (*Int. 36 –* senior public official).

A second aspect pertains to the horizontal interplay of municipalities with public and private actors in their jurisdictions. In Taranto, the search for consensus and the construction of political alliances have remained limited at the crucial moment of attempting to redefine the area’s development model after six decades of steel monoculture. Interviews confirm that the markedly institutional character of the ongoing process has overshadowed the so-called “civil society,” that is, the various associations and movements existing locally and, more generally, the citizens, who have been relegated to a gregarious role. Participation in defining the new long-term developmental strategy was constrained, limited to an informational nature; in some cases, it was more engaging, yet it occurred in selective circumstances, for example, when some professional associations (architects, engineers, accountants) were involved or at an individual level; rarely has it been an open and wide participation relating, for instance, to decisions and implementations (Cacciapaglia, 2025).

*Surely citizens were not actually involved in these processes; they received communication of these processes …. the consultations had an informative rather than participatory purpose ... despite the intensification of these events, these tools and these events proved to be totally self-referential. (Int. 20 – Startupper).*

The self-referential nature of the ongoing process appears paradoxical to many, given that the rhetoric surrounding it emphasizes inclusivity and intergenerational equity. If change is a process that leads to a different, better future, then among the main actors should be young people; however, in Taranto, this does not seem to be the case.

*The thing that should probably be worked on is the involvement of all (population) groups or at least of the most possible ones. (Int. 3 – Knowledge manager working for a public institution).*

The limited exercise of process-oriented leadership and the involvement of other actors are especially questionable, given that some local groups and associations had produced programmatic documents to contribute to the debate. These include “Piano Taranto” developed by Giustizia per Taranto together with other local environmental associations (Giustizia per Taranto, 2018; Boniburini and Esposito, 2025) and, more recently, “T.R.A.C.C.E.” (TRACCE, 2026), which builds upon Piano Taranto to propose an alternative vision of urban regeneration. Both documents call for overcoming the ambiguous stance of public institutions regarding the decarbonization of the former ILVA plant and argue that Taranto’s new developmental trajectory should be based on an adaptive regeneration program combining industrial heritage, creative districts, the energy transition and social innovation. Admittedly, the limited involvement of local social and environmental movements as active participants in the process can also be attributed to the fragmented and fragile nature of the local social fabric, shaped by the conflicts arising from the ILVA crisis. Finally, in this picture, the absence of initiative among private business actors is striking. They appear either unwilling or unable to contribute to leading the area’s renewal. Rather than articulating an autonomous development strategy, the business class seems to follow the choices and priorities set by public institutions. While weakened in their organizational and market power, with limited room for maneuver given ex-ILVA’s national relevance, trade unions have nevertheless been vocal in demanding a labor-driven transition that ensures quality jobs, workers’ protection and the continued presence of industry in Taranto.

The institutional approach therefore remained uncontested, despite notable and novel visions of the transition as a process of community social regeneration centered on civic infrastructures. As indicated by an interviewee:

*Some people understand transition simply as an economic transition when Taranto needs perhaps other types of transaction, mainly cultural. … What needs to be overcome is cultural and social poverty, in the sense of a lack of basic social services and poor social investments. I repeat many mean this transition as an energy transition …., personally I would prefer the transition to start from social works and services. (Int. 1 – Warehouse worker in a private company).*

An additional aspiration aims at the achievement of a collective dimension. As indicated:

*The right transition includes all these dimensions [environmental, technological, social] and moves away from a status quo… but this cannot be a personal goal, it must be a community goal. (Int. 38 – Expert of local development, Consultant).*

## Conclusion

The article has outlined the contours of the governance of the socio-economic transition of the industrial city of Taranto toward a greener, more sustainable future. From a theoretical viewpoint, the article provides new evidence on the mechanisms by which cities and their administrations might modify their development trajectories towards sustainability, and on the possible shortcomings associated with them; ultimately, it offers evidence on the complexities of multi-level governance dynamics. The article also contributes to the literature on industrial areas by drawing insights from the sustainable transition of the Taranto area, which remains one of the most paradigmatic cases in Europe and beyond.

Based on the original evidence, the article highlights the protagonism of local public institutions in the governance of the socio-economic transition in Taranto, namely, the Puglia Region and the Municipality of Taranto. After years of institutional deadlock, a process of diversification of the local economy has begun. Its reconfiguration also draws on symbolic assets including the area’s natural beauty and its ancient Greek heritage. Through a mix of ideational, political and economic mechanisms, local public institutions have adopted a post-industrial economic model and conveyed a vision of transition in Taranto in line with a green growth perspective, one that assumes that it is possible to foster economic growth and development while reducing environmental harm and using natural resources more sustainably.

While positively valuing the unprecedented effort to diversify the area’s economy and society, even though it has so far produced limited tangible impacts, the local public-led governance has been less successful in ensuring autonomous leadership and in incorporating the demands of different societal groups. Legal frameworks and power asymmetries have hindered autonomy from other institutional levels. Involvement has often taken the form of ritual consultations, managed in a hegemonic manner and never truly regarded as a resource; as a result, both the institutional innovation associated with the strategic planning, conceived as a means to build social capital and forge a new pact for the future, and local activism have been significantly weakened. In terms of the tension between work and health, the recent ruling by the Milan Court of Appeals, ordering the imminent closure of the heat treatment area at the ex-ILVA plant, opens a new, complex phase in the search for a solution to the industrial dispute. At the same time, local society is once again divided between those who see the ruling as doing what politicians have failed to do and those who fear a social implosion of the community.

If there is to be an ecological transformation, States, at their sub-national levels, are certainly key actors, with the capacity to enhance societal transformations by orchestrating and/or enabling multiple socio-technical transitions (including food, water, energy, transport, housing, etc.). No other institution can match the State’s regulatory capacity. Nor is there any other institution with the resources and financial transfer mechanisms to provide social welfare and address inequalities and injustices on the scale of States (Eckersley, 2021; Newell, 2026). Nonetheless, the capacity of local institutional actors to exercise leadership, establishing a “green economy” that delivers “green growth” and creates “green jobs” (Newell, 2026; Hudson, 2024), without incorporating larger social strata, risks becoming a new mantra without solid pillars, as it is an oversimplification of the belief that the restart of the economy on new bases will naturally bring with it the improvement of citizens’ living and working conditions. The experience of transition in Taranto hardly questions the issue of justice; even when it is explicitly required, as in the case of the JTF. The emerging evidence reveals an unproblematic acceptance of the prevailing mainstream paradigm and a lack of reflection on the long-lasting and multidimensional crisis that hit Taranto.

Further research is called to engage with the study of environmental governance at various levels and in various contexts by providing insight into how governance arrangements are being and could be developed to ensure progressive modernizations and democratic transformations, especially in industrial areas where difficulties, setbacks and discontents are likely to slow down or even impede such processes. Appreciating nature and the logic of action by State’s actors is crucial to both sustaining and critiquing contemporary State practices and to possibly searching for alternative visions. In this sense, one shares the position of some scholars (Routledge et al., 2018) who, recalling Wright’s work, advocate for a processual conception of the State that recognizes it as a terrain of possibility for *positive* social, economic and environmental change, constantly reworked by the interaction of actors and their strategies. This opens opportunities to include, for instance, grassroots mobilization and social movements and to engender just social formations in the context of climate change. This is not only a research avenue but also a necessity for shaping equitable and sustainable environmental governance practices and policy choices.

## Data Availability

The original contributions presented in the study are included in the article/supplementary material, further inquiries can be directed to the corresponding author/s.

## Footnotes

1 Such issues have also been of some relevance also in the eco-social literature (among others, Bonetti and Villa, 2023; Jessoula and Mandelli, 2022; Schwander and Fischer, 2025).

2 The rate of industrialization is measured as the ratio of industrial workers to the total population.

3 Having designated the ILVA plant as a national strategic asset, responsibility for managing its crisis and possible resolution was effectively shifted from local authorities to the national government. Industrial relations are also centrally managed.

4 The law has the following title: “Guidelines for the development, environmental sustainability and socio-economic cohesion of the area of Taranto.”

5 To this day, the port has retained its predominantly industrial character, serving the steel plant almost exclusively; however, some areas have been destined for containers transshipment.

6 This Strategic Plan sits withing the Recovery and Resilience Fund approved by the EU after the COVID-19 crisis.

7 Renzi (Italy’s former Prime Minister), Emiliano (former President of the Puglia Region), and Melucci (former Mayor of Taranto) belonged to the same center-left party, the Democratic Party.

8 The homogeneity of policy coalitions might become an important, general factor in their effectiveness if, for instance, the current proposed reform of the EU cohesion policy, which would return more power to the national rather than local levels, is approved.

9 Such a re-organization was promoted by the right-wing Minister Raffaele Fitto, later nominated as an EU Commissioner.

10 Programmi integrati di agevolazione (PIA) for medium enterprises and mini-PIA for micro and small enterprises provide financial resources for supporting innovation and production investments, favor digitalization and ecological transition, promote regional employment, especially for women, and support firms’ internationalization.
